# Supplementary material for: Identification of photoperiod-induced specific miRNAs in the adrenal glands of Sunite sheep (Ovis aries)
Source: Front Vet Sci. 2022 Jul 22;9:888207. doi: 10.3389/fvets.2022.888207 (PMC9354845; doi:10.3389/fvets.2022.888207)
Supplement: Supplementary file 1 [file Data_Sheet_1.ZIP › Supplementary file/miRDeep2 file.zip!/miRDeep2 file/Novel_Structure/LP42A1/Novel_40.pdf]

Provisional ID	:	NC_019460.2_7880
Score total	:	294.3
Score for star read(s)	:	3.9
Score for read counts	:	286.6
Score for mfe	:	2.1
Score for randfold	:	1.6
Score for cons. seed	:	
Total read count	:	574
Mature read count	:	456
Loop read count	:	0
Star read count	:	118

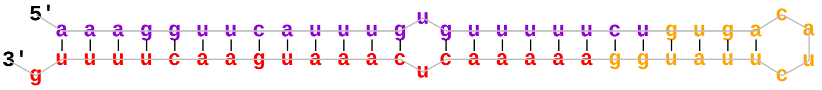

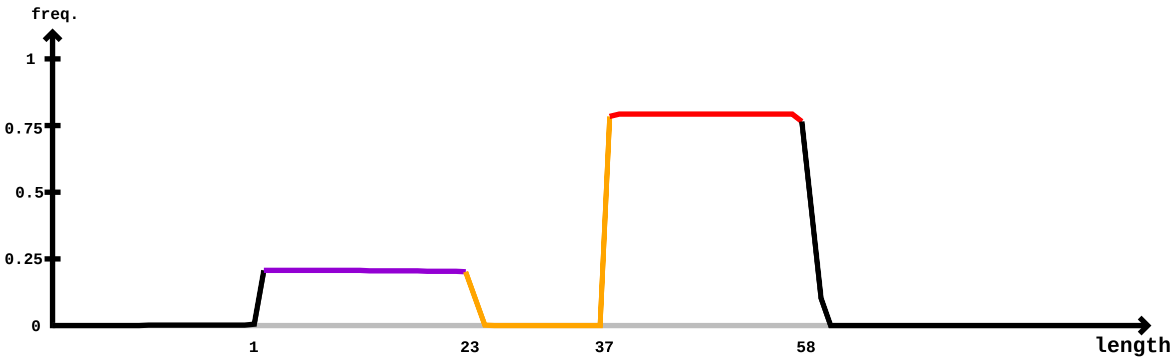

Star **Mature**

```
5'- aaauaauacuggguuggccaaaagguucauuuguguuuuucugugacaucuuauggaaaaacucaaaugaacuuuugggccaacccaaugaaaagcuccaaucccuucacuc  -3'   obs
    aaauaauacuggguuggccaaaagguucauuuguguuuuucugugacaucuuauggaaaaacucaaaugaacuuuugggccaacccaaugaaaagcuccaaucccuucacuc         exp
    .........((((((((((.(((((((((((((.((((((((((((....)))))))))))).))))))))))))).)))))))))).........................   reads   mm   sample
    ........Uggguuggccaaaagguucauu..................................................................................   1       1    seq
    ...................aaaagguucauuuguguuuuuc.......................................................................   1       0    seq
    ...................aaaagguucauuuguguuuuucu......................................................................   1       0    seq
    ....................aaagguucauuuguguu...........................................................................   1       0    seq
    ....................aaagguucauuuguguuuuucu......................................................................   114     0    seq
    ....................aaagguucauuuguguuuuucugu....................................................................   1       0    seq
    ........................................................aaaaacucaaaugaacuuuC....................................   2       1    seq
    ........................................................aaaaacucaaaugaacuuuu....................................   14      0    seq
    ........................................................aaaaGcucaaaugaacuuuug...................................   1       1    seq
    ........................................................aaaaacucaaaugaacuuuuU...................................   5       1    seq
    ........................................................aaaaacucaaaugaacCuuug...................................   3       1    seq
    ........................................................aaaaacucaaaCgaacuuuug...................................   1       1    seq
    ........................................................aaaaacucaaaugaUcuuuug...................................   1       1    seq
    ........................................................aaaaacucaaaugaacuuuuA...................................   31      1    seq
    ........................................................Gaaaacucaaaugaacuuuug...................................   1       1    seq
    ........................................................aGaaacucaaaugaacuuuug...................................   4       1    seq
    ........................................................aaaaacucaaaugaacuuuug...................................   271     0    seq
    ........................................................aaaaacucaaaugaacuuuuUg..................................   9       1    seq
    ........................................................aaaaacucaaaugaacuuuugg..................................   49      0    seq
    ........................................................aaaaGcucaaaugaacuuuugg..................................   1       1    seq
    ........................................................aaaaacucaaaugaacuuuuggU.................................   47      1    seq
    ........................................................aaaaacucaaaugaacuuuuggg.................................   4       0    seq
    ........................................................aaaaacucaaaugaacuuuuggC.................................   7       1    seq
    .........................................................aaaacucaaaugaacuuuug...................................   1       0    seq
    .........................................................aaaacucaaaugaacuuuuUg..................................   3       1    seq
    .........................................................aaaacucaaaugaacuuuuggC.................................   1       1    seq
```
